# Exploring the Antineoplastic Properties of the Lebanese *Jania rubens* Against Colorectal Cancer

**DOI:** 10.3390/metabo15020090

**Published:** 2025-02-02

**Authors:** Mariam Rifi, Zeina Radwan, Nouha Sari-Chmayssem, Rayan Kassir, Ziad Fajloun, Abir Abdel Rahman, Marwan El-Sabban, Corinne Prévostel, Zeina Dassouki, Hiba Mawlawi

**Affiliations:** 1 Laboratory of Applied Biotechnology (LBA3B), AZM Center for Research in Biotechnology and its Applications, Doctoral School for Sciences and Technology, Lebanese University, Tripoli 1300, Lebanon; maryamrifi8@gmail.com (M.R.); n.sari@ul.edu.lb (N.S.-C.); rayan.kasir@bau.edu.lb (R.K.); ziad.fajloun@ul.edu.lb (Z.F.); 2 Department of Anatomy, Cell Biology, and Physiological Sciences, Faculty of Medicine, American University of Beirut, Beirut 1107-2020, Lebanon; zar04@mail.aub.edu (Z.R.); me00@aub.edu.lb (M.E.-S.); 3 Faculty of Public Health III, Lebanese University, Tripoli 1310, Lebanon; 4 Faculty of Sciences 3, Lebanese University, Michel Slayman Tripoli Campus, Ras Maska 1352, Lebanon; 5 Department of Medical Laboratory Sciences, Faculty of Health Sciences, University of Balamand, Beirut 55251, Lebanon; abir.abdelrahman@balamand.edu.lb; 6 IRCM (Montpellier Cancer Research Institute), University of Montpellier, Inserm, ICM (Montpellier Regional Cancer Institute), 34298 Montpellier, CEDEX 5, France; corinne.prevostel@inserm.fr

**Keywords:** colorectal cancer, red algae, *Jania rubens*, antineoplastic effect, polysaccharide extract, FT-IR methods, cytotoxicity

## Abstract

Background/Objective: Colon cancer poses a significant health burden, with current treatments often associated with severe side effects and limited effectiveness for some patients. Natural products are gaining interest as adjuvant therapies, potentially reducing side effects and improving responses to conventional treatments. We previously highlighted the potent antineoplastic effects of organic extracts derived from the Lebanese red algae *Jania rubens*. This study, investigated the anticancer activities of polysaccharide, protein, and lipid extracts from *J. rubens*, which may serve as adjuvant therapies to enhance conventional treatments. Methods: we employed colorimetric assays, wound healing assays, and cell cycle analysis to evaluate the anticancer activities of the extracts. The polysaccharide extract was characterized for sulfate content and structure using barium chloride-gelatin and FT-IR methods. Results: All *J. rubens* extracts exhibited significant anticancer effects, with the polysaccharide extract showing particularly strong cytotoxicity, apoptosis induction, and antiproliferative and anti-migratory activities. Conclusion: These findings confirm that *J. rubens* is a source of bioactive compounds with anticancer potential. Further investigations are needed to elucidate the molecular pathways targeted by *J. rubens* extracts in cancer cells.

## 1. Introduction

Cancer represents a major risk to global health, standing as one of the leading causes of mortality and morbidity around the world [1,2,3]. Developing countries have experienced a significant increase in cancer incidence compared to Western countries. This rise in cancer incidence may be linked to the socioeconomic status of the world population, along with an increase in risk factors related to globalization and economic growth [4]. Increased intake of processed foods, sedentary lifestyles, and rising obesity rates are among these risk factors [5].

Lebanon has experienced a high incidence of all cancer subtypes compared to other Arab countries [6,7,8,9]. According to the Lebanese National Cancer Registry, colorectal cancer (CRC) has emerged as one of the most common and fatal cancers due to significant incidence rates [10]. To date, surgery, chemotherapy, radiotherapy, and targeted therapy are the primary treatment options for advanced stages of cancer. However, chemotherapy is often associated with serious adverse effects and drug intolerance [11]. Due to the narrow therapeutic index of many conventional therapies, there is growing interest in alternative remedies derived from natural-based sources, which are valued for their safety and low toxicity [12].

Recently, marine compounds, particularly macroalgae, have been identified as a valuable source of bioactive molecules, showing promise for the development of new therapeutic agents [13,14,15,16,17]. Remarkably, there is growing interest in investigating the medicinal properties of natural seaweed as a therapeutic adjuvant in cancer treatment, aiming to improve the effectiveness of anticancer treatments while protecting healthy tissues from the harmful effects of chemotherapy. However, research on the bioactive substances derived from seaweeds and their anticancer potential is still in its infancy [5,18,19,20,21].

Seaweeds have become a significant source of bioactive compounds with various biological activities, including anti-inflammatory, anti-coagulant, anti-obesity, antibacterial, anti-viral, anti-fungal, antioxidant, and antitumoral properties. Numerous studies have characterized their chemical composition, which includes polysaccharides, carbohydrates, polyphenols, lipids, fatty acids, sterols, peptides, amino acids, photosynthesis pigments, vitamins, as well as minerals [22]. In addition, seaweeds contain peptides, proteins, steroids, phlorotannins, fucoidan sugars, mannitol, terpenoids, and glycolipids as secondary metabolites [23]. Among the wide variety of seaweeds, Rhodophyta, also known as red algae, is the most primitive group in the phylogenetic tree, comprising around 6000 species [17]. Red algae are distinct from terrestrial medicinal plants due to their chemical composition, particularly high levels of proteins [22,24]. They are also rich in phycobiliproteins, which exhibit a variety of biological activities, including antitumor [25,26], anti-hypertension [27], and anti-coagulant effects [28]. In Asia, red seaweed is widely consumed as a health-enhancing dietary supplement and nutraceutical, known to reduce disease risk, enhance prebiotic effects, and improve digestion [22]. Global interest in the nutritional value of red seaweed is growing, leading to a dramatic increase in its demand on the world market [28]. One of the most alluring qualities of red seaweed as a food and therapeutic substance is its richness in biologically active polysaccharides, which constitute 40 to 50% of the algal dry weight and are primarily found in the cell walls. These polysaccharides are widely used in the pharmaceutical industry for their gelling and thickening properties [26]. Notably, sulfated galactan, including porphyrans, carrageenans, and agars, are the most relevant and exploited compounds present in the cell wall of red seaweed [26]. Many studies have attributed the biological activity of red algae to sulfated polysaccharides, particularly carrageenans [29,30], which have shown promising effects in the prevention and treatment of some malignancies [28,31]. Carrageenans, linear sulfated polysaccharides having a structure that alternates between 3-linked ß-D-galactose and 4-linked α-D-galactose, are classified into three sub-groups: kappa (j), iota (i), and lambda (k)) according to their sulfation degree, solubility, and gelling properties.

Red algae, which are abundant along the Lebanese coast, have been poorly investigated, despite their health benefits. *Jania rubens* has recently been studied by Rifi et al., demonstrating antioxidant and antitumor activities against colorectal cancer cells [32]. In this study, we prepared extracts of polysaccharides, proteins, and lipids from *J. rubens* and examined their anti-migratory and antiproliferative activities to assess their clinical relevance in treating colorectal cancer.

## 2. Materials and Methods

### 2.1. Collection of Macroalgae

At a depth of 2–3 m, samples of *J. rubens* were obtained from the Mediterranean coast of northern Lebanon. A fine powder was made from freshly rinsed seaweed that had been air-dried at room temperature. The herbarium voucher of *J. rubens* (AZM-1105) is retained at The Doctoral School of Science and Technology, Lebanese University.

### 2.2. Preparation of Extract

#### 2.2.1. Lipids Extraction

Fifty grams of dried and milled *J. rubens* was extracted with 700 mL of chloroform/hexane (2:1). The mixture was agitated overnight at room temperature and then filtered. The resulting supernatant containing lipids was evaporated under reduced pressure using a rotary evaporator, yielding 10 mg of lipids. The residue obtained from this extraction was subsequently used for protein extraction.

#### 2.2.2. Proteins Extraction

At 40 °C, 200 mL of ultrapure water was added to the residue from the previous extraction phase. After stirring the mixture for 24 h at 40 °C, it was filtered and combined with 20 mL of ultrapure water at 60 °C. Then, 1 mL of zinc sulfate (1 M) and barium hydroxide (1 M) was added, and the mixture was agitated for a few minutes to precipitate the proteins. Following this, the mixture was centrifuged for 10 min at 4 °C at 5000× *g,* and the protein extract-containing pellet was lyophilized, yielding 25 mg of proteins. The supernatant was reserved for polysaccharide extraction.

#### 2.2.3. Polysaccharides Extraction

The supernatant obtained from protein extraction was filtered to remove any residual proteins, and the filtrate was mixed with 50 mL of ultrapure water. The mixture was centrifuged twice at 4 °C and 5000× *g*. The supernatant that resulted was lyophilized to extract polysaccharides, yielding 300 mg of polysaccharides.

### 2.3. Total Sulfate Content and Fourier Transform Infrared Spectroscopy (FT-IR) Analysis

The sulfate concentration was measured turbidimetrically using potassium sulfate as a reference and the barium chloride/gelatin technique. The Fourier transform infrared spectroscopy (FT-IR) spectrum of carrageenans was obtained using a Thermo Nicolet instrument. Using a mortar, the polysaccharide sample was combined with anhydrous potassium bromide (KBr). A KBr disc was formed by compressing the mixture into 1 mm. A total of 32 scans of the KBr disc were conducted across the wave number range of 500–4000 cm^−1^ [33,34].

### 2.4. Cell Lines and Culture Conditions

The colon cancer cell lines HT-29 and HCT-116 were obtained from the American Type Culture Collection (ATCC). The cells were kept in DMEM at 37 °C in a humidified incubator with 95% air and 5% CO_2_. Then, 10% heat-inactivated fetal bovine serum and 1% penicillin-streptomycin (100 U·mL^−1^) were added to the medium.

### 2.5. MTT Cell Viability Assay

After being seeded in 96-well plates, the cells were allowed to adhere overnight. Once they reached 80% confluence, various concentrations of *J. rubens* polysaccharide and protein extracts (100–250–500 and 750 μg·mL^−1^) were applied. At 24 h and 48 h, the treatments were removed, and then the cells were incubated with MTT for 4 h at 37 °C in the dark. Untreated cells represented the control with 100% viability. Mean absorbance readings were reported as a percentage of viability compared to the control with absorbance measured at 570 nm using an ELISA microplate reader.

### 2.6. Trypan Blue Test

In a 24-well plate, colon cancer cells (HT-29 and HCT-116) were seeded at a density of 2 × 10^4^. Only the most active extracts—polysaccharide and protein extracts—were examined for cytotoxicity using the trypan blue assay based on MTT results. Both treated and untreated cells were washed, detached with trypsin, and stained with trypan blue (0.4%) after 24 and 48 h. A hemocytometer was used to count the amount of living cells versus dead cells under a light microscope. Each determination was performed in triplicate.

### 2.7. Wound-Healing Scratch Assay

A 24-well plate was seeded with HCT-116 and HT-29 cells, which were allowed to grow for 24 h. Once 90% confluence was achieved, a sterile 200 μL tip was used to create a scratch wound. Following two rounds of washing with 1XPBS (phosphate buffer solution), cells were incubated with varying doses of the algal extracts (0, 100, 250, 500, and 750 µg·mL^−1^). After 0, 6, and 24 h of treatment, pictures of the wounds were captured using a digital camera and a light microscope. The ImageJ analysis program was utilized to determine the wounds’ surface area.

### 2.8. Cell Cycle Analysis

HCT cells were seeded in a 24-well plate, cultured until reaching 80% confluency, then treated with different concentrations of polysaccharide extracts for 24 h. The media containing the dead cells was transferred to a 15 mL conical tube, and the attached living cells were detached by trypsinization within the same tube. The resulting mixture was then centrifuged to obtain a pellet. The pellet was washed with cold 1X PBS, fixed with 70% cold ethanol, and stored at −20 °C. Then, cells were washed with cold PBS and incubated for one hour with 100 μL DNase-free RNase A (200 μg/mL) before staining with 1 mg/mL of propidium iodide for 15 min. Using flow cytometry, the intensity of the fluorescence was determined with a Guava EasyCyte8 flow cytometer.

### 2.9. Chemicals

The reagents used in this study included sulfuric acid (Sigma-Aldrich; St. Louis, MO, USA) and nitric acid (Sigma-Aldrich; St. Louis, MO, USA). Hydrochloric acid, glucose, zinc sulfate, barium hydroxide, and potassium acetate laboratory chemicals—Lebanon). Bovine serum albumin (Sigma-Aldrich; 9048-46-8; St. Louis, MO, USA), acetone (Supelco PA, USA), dichloromethane (Sigma-Aldrich; St. Louis, MO, USA), methanol (Sigma-Aldrich; St. Louis, MO, USA), Folin–Ciocalteu phenol (Sigma-Aldrich; CAS 47641; St. Louis, MO, USA), ethanol (Sigma-Aldrich; St. Louis, MO, USA), aluminum chloride (Sigma-Aldrich; St. Louis, MO, USA), 2,2-diphenyl-1-picrylhydrazyl (Sigma-Aldrich; St. Louis, MO, USA), quercetin (Sigma-Aldrich), Dulbecco’s Modified Eagle’s Medium (Sigma-Aldrich; D5796; St. Louis, MO, USA), fetal bovine serum (Sigma-Aldrich; F9665; St. Louis, MO, USA), phosphate-buffered saline (Sigma-Aldrich, 806552; St. Louis, MO, USA), penicillin-streptomycin (Sigma-Aldrich, P4333; St. Louis, MO, USA), 3-(4,5-dimethylthiazol-2-yl)-2,5-diphenyltetrazolium bromide (Sigma-Aldrich; M56655; St. Louis, MO, USA), trypsin (Sigma Aldrich; T3924; St. Louis, MO, USA), and propidium iodide (Sigma-Aldrich; P4170; St. Louis, MO, USA).

### 2.10. Statistical Analysis

GraphPad Prism 7 software by Dotmatics (version 7.0, Boston, MA, USA) was used to conduct all statistical analyses *(t*-test, one-way ANOVA, and two-way ANOVA). Values below 0.01 (** *p* < 0.01), 0.001 (*** *p* < 0.001), and 0.0001 (**** *p* < 0.0001) were regarded as very significant, whereas probability values below 0.05 (* *p* < 0.05) were regarded as significant. The means ± SD of the specified set of experiments are used to express the quantitative results.

## 3. Results

### 3.1. The Cytotoxic Activity of J. rubens Extracts on Human Colon Cancer Cells

#### 3.1.1. Polysaccharide Extracts Reduce the Viability of HCT-116 Cells and HT-29 Cells

**Figure 1:**
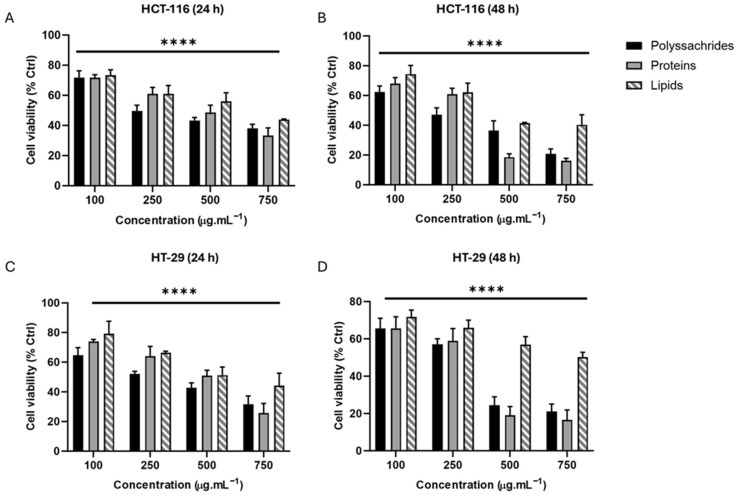
Effect of polysaccharides, proteins, and lipids extracts on colon cancer cell lines viability. HCT-116 and HT-29 were seeded and treated with various doses (100, 250, 500, and 750 µg/mL) in 96-well plates. HCT-116 and HT-29 cell viability was measured after 24 h (**A**,**C**) and 48 h treatment (**B**,**D**) by using the MTT tetrazolium reduction assay. The percentage of cell viability was calculated relative to the control value (100% viability). The mean ± SD (*n* ≥ 3) is used to demonstrate the results. **** *p* < 0.0001.

The cytotoxicity activity of polysaccharide extracts on HT-29 and HCT-116 colon cancer cells was investigated using an MTT assay. HT-29 and HCT-116 cell lines were subjected to varying concentrations of extracts (ranging from 100 to 750 µg/mL) at two time points (24 h and 48 h). A substantial reduction in cellular viability (*p* < 0.0001) was detected for both cell lines at all concentrations of polysaccharide extract, demonstrating dose and time cytotoxicity (Figure 1). A concentration of 750 µg/mL from polysaccharide extract reduced the viability of cells to (38 ± 5.5%) in HCT-116 and (31 ± 5.8%) in HT-29 at 24 h treatment, compared to the non-treated control. After 48 h of treatment, cell viability further reduced to (21 ± 4.01%) in HT-29 and (20.66 ± 3.4%) in HCT-116 at the same concentration. As shown in Table 1, HT-29 was more sensitive than HCT-116 cells to polysaccharide treatment (IC_50_ = 272.1 µg/mL versus 324.7 µg/mL). The results were validated using a trypan blue assay. Therefore, polysaccharides demonstrate a strong cytotoxic property in both HT-29 and HCT-116 cells.

#### 3.1.2. Protein Extracts Reduce the Viability of HT-29 and HCT-116 Cells

The administration of protein extract over durations of 24 h and 48 h led to a statistically significant reduction in cellular viability (*p* < 0.0001 when compared to the control) in a dose- and time-dependent manner for the two cell lines examined (Figure 1). HCT-116 cells showed a decrease in cell viability from (75.99 ± 3.25%) at 100 µg/mL to (38.35 ± 5.76%) at 750 µg/mL protein extract. In addition, HCT-116 cell viability was reduced to 33.35 ± 5.76% after 24 h and to 16.32 ± 2.3% after 48 h of treatment. For HCT-116 and HT-29 cells, the IC50 values were 389.4 µg/mL and 382.8 µg/mL, respectively, indicating that protein extracts exert a similar cytotoxic effect on both CRC cell lines (Table 1).

#### 3.1.3. Lipid Extracts Reduce the Viability of HT-29 and HCT-116 Cells

Similar to polysaccharide and protein extracts, lipid extracts significantly decreased colon cancer cell viability *p* < 0.0001 (Figure 1). However, the results showed a dose-dependent effect without a clear time-dependent response. Upon treatment of the HCT-116 cells with 100 µg/mL of lipid extract, cell viability was reduced to 73.25 ± 5.32%, decreasing further to 43.25 ± 4.24% with 750 µg/mL treatment. The IC_50_ values were 627.7 µg/mL and 588.5 µg/mL for HCT-116 and HT-29, respectively (Table 1).

Together, these results demonstrate a potent cytotoxic effect of *Jania*’s extracts on colon cancer cells, with polysaccharide and protein extracts showing higher efficacy compared to lipid extracts.

### 3.2. The Anti-Migratory Effect of J. rubens Extracts on Colon Cancer Cell Lines

#### 3.2.1. *J. rubens* Polysaccharide Extracts Prevent the Migration of Colon Cancer Cells

**Figure 2:**
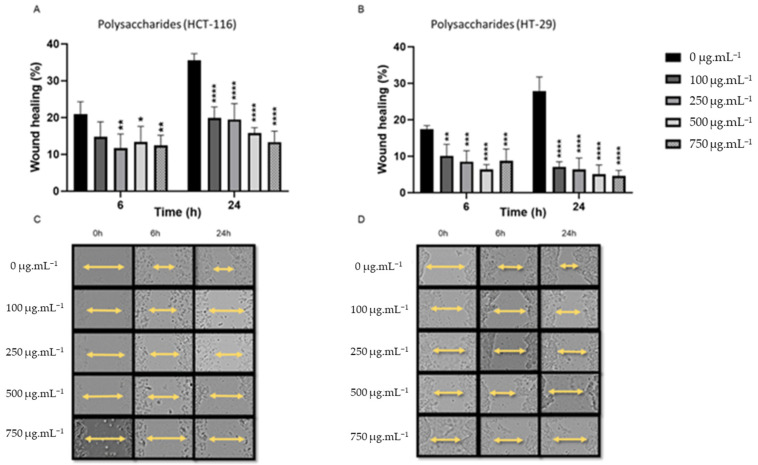
Wound healing assay demonstrating that a range of concentrations from 100 to 750 µg/mL of polysaccharide extracts significantly reduce the migrating properties of HCT-116 and HT-29 cells following treatment duration of 6 and 24 h. (**A**,**B**) Polysaccharides treatment of HCT-116. (**A**) Percentage of wound healing at 0, 6, and 24 h. (**B**) Images of the scratch were obtained and documented at 0, 6, and 24 h. (**C**,**D**) Polysaccharides treatment of HT-29. (**C**) Percentage of wound closure was assessed at time intervals of 0, 6, and 24 h. (**D**) Images of the scratch were obtained and documented at 0, 6, and 24 h. Representative images were acquired utilizing a Leica microscope. The data were expressed as mean ± standard deviation, with a sample size of n = 3. * *p* < 0.05, ** *p* < 0.01, *** *p* < 0.001, and **** *p* < 0.0001 vs. control group.

Polysaccharide extracts were evaluated for their ability to inhibit the migratory potential of human HCT-116 and HT-29 cells by using the wound healing scratch assay [32]. As shown in Figure 2, polysaccharide extracts significantly reduced the wound closure for both cell lines in a time- and dose-dependent manner (0.01< *p* < 0.00001). After 24 h, HCT-116 cells treated with 100 µg/mL polysaccharides revealed a wound closure rate of 19.87 ± 3% compared to control 35.55 ± 1.8%. This ratio further decreased to 13% with 750 µg/mL polysaccharide extracts. Likewise, 750 µg/mL treatment dramatically decreased the gap-closing percentage of HT-29 cells to 4.57% after 24 h, which is seven times lower than the control (Table 2). Notably, polysaccharide extracts also significantly decreased the wound closure at an early time point (6 h) for both cell lines. These results indicate that polysaccharide extracts exert a potent anti-migratory effect on colon cancer cells.

#### 3.2.2. *J. rubens* Protein Extracts Prevent the Migration of Colon Cancer Cells

**Figure 3:**
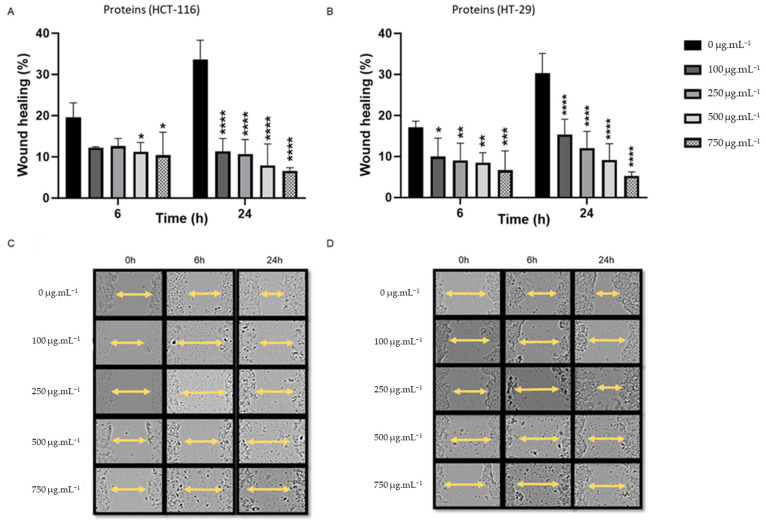
Wound healing assay demonstrating that a range of concentrations from 100 to 750 µg/mL of protein extracts significantly reduce the migrating properties of HCT-116 and HT-29 cells following treatment duration of 6 and 24 h. (**A**,**B**) Protein treatment of HCT-116. (**A**) Percentage of wound healing at 0, 6, and 24 h. (**B**) Images of the scratch were obtained and documented at 0, 6, and 24 h. (**C**,**D**) Protein treatment of HT-29. (**C**) Percentage of wound closure was assessed at time intervals of 0, 6, and 24 h. (**D**) Images of the scratch were obtained and documented at 0, 6, and 24 h. Representative images were acquired utilizing a Leica microscope. The data were expressed as mean ± standard deviation, with a sample size of *n* = 3. * *p* < 0.05, ** *p* < 0.01, *** *p* < 0.001, and **** *p* < 0.0001 vs. control group.

As shown in Figure 3, cells treated with increasing concentrations (100–750 µg/mL) of protein extracts also exhibited a significant dose- and time-dependent anti-migration effect (0.01 < *p* < 0.00001). In comparison to 33% for the control, the percentage of HCT-116 wound healing at 24 h dropped to 11.3% and 6.6% following 100 µg/mL and 750 µg/mL treatment, respectively. For HT-29, the wound closure rate was 15.3% at 100 µg/mL treatment, decreasing to 5.2% at 750 µg/mL treatment compared to 30% for the control (Table 3). Again, protein extract significantly reduced the gap closure rate early (6 h) in both cell lines.

### 3.3. Cell Cycle Disruption of Colon Cancer Cells by Polysaccharide Extracts

**Figure 4:**
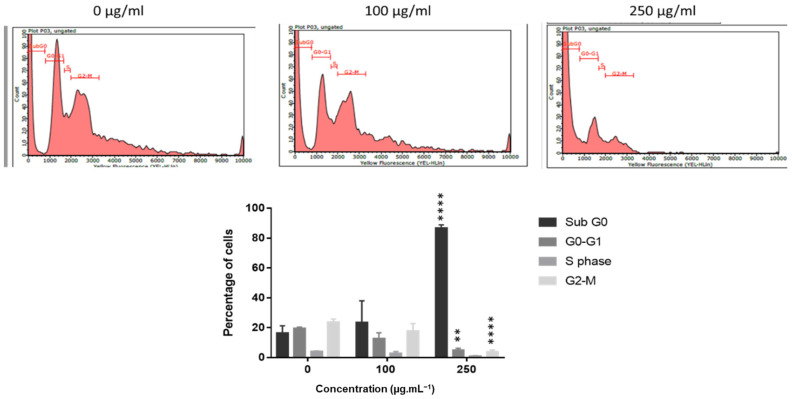
Polysaccharide extract increased the sub-G0 population of HCT-116 cells. Flow cytometry analysis demonstrates that polysaccharide extracts of *J. rubens* increases the subG1 population and decreases G0/G1 and G2/M populations of HCT-116 cells after treatment with 250 μg/mL polysaccharide extracts for 24 h. Data values are represented as mean ± SD (*n* = 3). ** *p* < 0.01 **** *p* < 0.0001.

Since *J. rubens* polysaccharide extracts were the most efficient for inhibiting colon cancer cell growth, we performed cell cycle analysis on HCT-116 cells using flow cytometry. As shown in Figure 4, polysaccharide extracts induced significant changes in the cell cycle of HCT-116 cells. Untreated cells (control) displayed a standard cytogram of a population of diploid cells, while cells treated with 250 μg/mL of polysaccharide extracts for 24 h accumulated significantly in the sub-G0 phase, with 86.69% compared to 16.48% for the control (over five times more). Following treatment with 250 μg/mL polysaccharide extracts, the number of cells in the G0/G1 phase dropped significantly, reaching 4.86% as opposed to 19.61% for control cells. Furthermore, following treatment with 250 μg/mL polysaccharide extracts, the number of cells in the G2/M phase dropped by six times (Figure 4).

### 3.4. FT-IR Analysis of Polysaccharide Extract and Total Content of Sulfate

**Figure 5:**
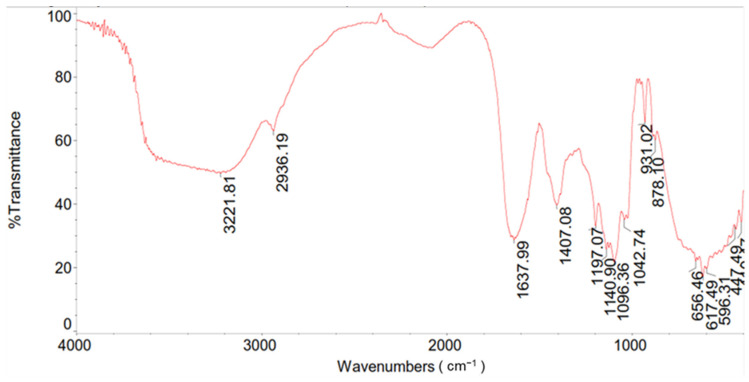
FT-IR analysis of polysaccharides extracted from the red algae *J. rubens*.

We have demonstrated that *J. rubens*-extracted polysaccharides possess antiproliferative and anti-migratory properties. To further characterize the composition of the polysaccharide extract, we utilized Fourier transform infrared spectroscopy (FT-IR). The IR method only needs a few milligrams of sample and is a quick and non-destructive procedure. Figure 5 shows the FT-IR spectrum in detail. A broad band of vibration between 3221 and 3545 cm^−1^ is due to the hydroxyl groups’ (OH) stretching mode, while the C-H stretching vibrations are represented by the band at 2936 cm^−1^, which are the characteristic absorption peaks of sugar. In addition, the bands at 1197 cm^−1^ and 878 cm^−1^ are indicative of the ester sulfate groups in the C4 position of galactose units. The bands at 931 and 1042 cm^−1^ reflect the stretching vibration of the C-O-C of 3,6 anhydrous-galactose units [35,36,37].

This spectrum aligns with the typical spectrum of carrageenan, as previously documented in the literature [35,36,37]. Furthermore, we demonstrated that the extracted polysaccharide has a high amount of sulfate content, measured at 38%.

## 4. Discussion

Emerging drug resistance and systemic adverse effects during cancer therapy encourage active search for novel therapeutic agents [38]. A significant opportunity for research lies within the underused bioactive potential of natural products, which could significantly enhance the efficacy and quality of treatments [23]. For centuries, traditional medicine, particularly in Asian cultures, has utilized seaweed to treat various ailments [39]. Notably, red algae are gaining significant interest as a natural source of bioactive compounds with potential health benefits [14,22]. Among these bioactive compounds, sulfated polysaccharides (SPs), particularly carrageenan, have received considerable attention, with numerous studies highlighting their diverse bioactivities, including anti-coagulant, anti-viral, antioxidant, and antitumoral effects, with immune-modulatory and cholesterol-lowering properties [22,23].

In a previous study, we investigated the antineoplastic effects of the Lebanese red algae *Jania rubens* [32]. The initial analysis revealed a composition of 14.5% carbohydrates, 11.3% proteins, and 4.5% fats. The most abundant minerals were magnesium (24 mg/g) and calcium (33 mg/g). Notably, all *J. rubens* extracts exhibited intense antioxidant activity, and organic extracts (dichloromethane/methanol DM and methanol M) showed greater antitumor activity against the human colon cancer cell lines HCT-116 and HT-29 compared to aqueous extracts. These findings suggest that high-temperature Soxhlet extraction is more effective in concentrating bioactive compounds with antitumor activity from *J. rubens*.

This study further investigated the anticancer properties of the Lebanese *J. rubens*. The isolated components, particularly polysaccharides and protein extracts, showed time- and dose-dependent cytotoxic effects on the cancer cell lines tested. Polysaccharide extracts proved to be the most potent, causing cell death, arresting the cell cycle, and blocking cell migration in HCT-116 and HT-29 colon cancer cells. Supporting this, studies on other red seaweeds have shown that polysaccharides can trigger cytotoxicity, induce apoptosis, or cause cell cycle arrest in many cancer cell lines like A549 [29], THP-1 [40], HepG2 [41], HeLa [42,43], and MCF-7 [44]. Furthermore, *J. rubens* polysaccharides were found to increase the expression of the cell death-inducing genes Bax, caspase-8, and P53 in CaCo-2 cells while up-regulating the level of expression of caspase-3 and downregulating the level of expression of Bcl-2 in MCF7 cells [45].

The strong antiproliferative and anti-migratory potential observed with *J. rubens* polysaccharide extract may be attributed to its richness in highly sulfated polysaccharides (SPs), particularly carrageenan [23,46]. It has been extensively shown that the degree of sulfation (DS), the molecular weight, the monosaccharide content, and the glycosidic linkage are some of the structural characteristics of polysaccharides that are directly linked to their biological activity [33,47]. Previous research has also demonstrated the anticancer properties of various types of carrageenan. For instance, κ-carrageenan has been shown to induce apoptosis in colon cancer cells (HCT-116) through mechanisms like ROS production, caspase-3 activation, and XIAP downregulation [43]. Additionally, certain types of carrageenan, such as κ-carrageenan and λ-carrageenan, have been reported to slow cell cycle progression in a variety of cancer cell lines (HeLa cells and HepG2) [48,49,50,51]. To further analyze the composition and the chemical structure of the components of *J. rubens* polysaccharide extracts, we employed FT-IR and quantified the sulfate content of the polysaccharide extract using the turbidimetric method with the barium-chloride gelatin reagent. The high sulfate content in the backbone of these polysaccharides, particularly carrageenan, aligns with previous research suggesting that the inhibition of colon cancer cell growth and migration by polysaccharide extracts from the Lebanese *J. rubens* is likely due to their sulfated nature [52,53].

## 5. Conclusions

Our results demonstrate that extracts from Lebanese *J. rubens* exhibit promising anticancer activities, suggesting their potential future use as adjuvants to enhance the efficacy of traditional chemotherapies. However, further studies are needed to elucidate the precise underlying biological activities. Additionally, the encouraging results warrant further analysis of the chemical composition of *J. rubens* extracts and investigation into the benefits of potentially using these extracts in combination with established therapies [32].

## Figures and Tables

**Table 1:** Cytotoxic activity of different extracts of *J. rubens* on CRC cell lines. Data are extracted from Figure 1 and reported as mean ± SD (n = 3).

In Vitro Cytotoxicity IC_50_ (µg/mL)		
Extracts	HCT-116	HT-29
Polysaccharides	324.7	272.1
Proteins	389.4	382.8
Lipids	588.5	627.7

**Table 2:** Percentage of wound repair in response to polysaccharide treatment at 24 h. Data are extracted from Figure 2 and reported as mean ± SD (*n* = 3).

Wound Healing % (Polysaccharides) 24 h		
Concetration (µg/mL)	HCT-116	HT-29
0	35.55	27.88
100	19.87	7.12
250	19.48	6.36
500	15.78	5.07
750	13.35	4.57

**Table 3:** Percentage of wound repair in response to protein treatment at 24 h. Data are extracted from Figure 3 and reported as mean ± SD (*n* = 3).

Wound Healing % (Proteins) 24 h		
Concetration (µg/mL)	HCT-116	HT-29
0	33.67	30.28
100	11.35	15.38
250	10.72	12.04
500	7.90	9.18
750	6.60	5.28

## Data Availability

The original contributions presented in this study are included in the article. Further inquiries can be directed to the corresponding authors.

## References

[1] Fitzmaurice C., Allen C., Barber R.M., Barregard L., Bhutta Z.A., Brenner H., Dicker D.J., Chimed-Orchir O., Dandona R., Dandona L. (2017). Global, regional, and national cancer incidence, mortality, years of life lost, years lived with disability, and disability-adjusted life-years for 32 cancer groups, 1990 to 2015: A systematic analysis for the global burden of disease study. JAMA Oncol..

[2] Khachfe H.H., Salhab H.A., Fares M.Y., Khachfe H.M. (2019). Current state of hypertrophic cardiomyopathy clinical trials. Glob. Heart.

[3] Bray F., Laversanne M., Sung H., Ferlay J., Siegel R.L., Soerjomataram I., Jemal A. (2024). Global cancer statistics 2022: Globocan estimates of incidence and mortality worldwide for 36 cancers in 185 countries. CA Cancer J. Clin..

[4] Wild C.P. (2019). The global cancer burden: Necessity is the mother of prevention. Nat. Rev. Cancer.

[5] Mabate B., Daub C.D., Pletschke B.I., Edkins A.L. (2023). Comparative analyses of fucoidans from south african brown seaweeds that inhibit adhesion, migration, and long-term survival of colorectal cancer cells. Mar. Drugs.

[6] Khachfe H.H., Rahal Z., Sammouri J., Kheil M., Baydoun H., Chatila D., Dirawi H., Fouad F.M. (2020). Cancer in lebanon: A review of incidence rates from 2008 to 2015 and projections till 2025. South Asian J. Cancer.

[7] Salhab H.A., Fares M.Y., Khachfe H.H., Khachfe H.M. (2019). Epidemiological study of lung cancer incidence in lebanon. Medicina.

[8] Fares M.Y., Salhab H.A., Khachfe H.H., Khachfe H.M. (2019). Breast cancer epidemiology among lebanese women: An 11-year analysis. Medicina.

[9] Charafeddine M.A., Olson S.H., Mukherji D., Temraz S.N., Abou-Alfa G.K., Shamseddine A.I. (2017). Proportion of cancer in a middle eastern country attributable to established risk factors. BMC Cancer.

[10] Lakkis N.A., El-Kibbi O., Osman M.H. (2021). Colorectal cancer in lebanon: Incidence, temporal trends, and comparison to regional and western countries. Cancer Control.

[11] Cotas J., Pacheco D., Gonçalves A.M., Silva P., Carvalho L.G., Pereira L. (2021). Seaweeds’ nutraceutical and biomedical potential in cancer therapy: A concise review. J. Cancer Metastasis Treat..

[12] Huang M., Lu J.-J., Ding J. (2021). Natural products in cancer therapy: Past, present and future. Nat. Prod. Bioprospecting.

[13] Saadaoui I., Rasheed R., Abdulrahman N., Bounnit T., Cherif M., Al Jabri H., Mraiche F. (2020). Algae-derived bioactive compounds with anti-lung cancer potential. Mar. Drugs.

[14] Qiu S.-M., Aweya J.J., Liu X., Liu Y., Tang S., Zhang W., Cheong K.-L. (2022). Bioactive polysaccharides from red seaweed as potent food supplements: A systematic review of their extraction, purification, and biological activities. Carbohydr. Polym..

[15] Al Monla R., Salma Y., Kouzayha A., Gali-Muhtasib H., Dassouki Z., Mawlawi H. (2021). Antioxidative, cytotoxic, and anti-metastatic potentials of laurencia obtusa and ulva lactuca seaweeds. Asian Pac. J. Trop. Biomed..

[16] Mehra R., Bhushan S., Bast F., Singh S. (2019). Marine macroalga caulerpa: Role of its metabolites in modulating cancer signaling. Mol. Biol. Rep..

[17] Yun C.W., Kim H.J., Lee S.H. (2019). Therapeutic application of diverse marine-derived natural products in cancer therapy. Anticancer. Res..

[18] Han N., Li J., Li X. (2022). Natural marine products: Anti-colorectal cancer in vitro and in vivo. Mar. Drugs.

[19] Barbalace M.C., Malaguti M., Giusti L., Lucacchini A., Hrelia S., Angeloni C. (2019). Anti-inflammatory activities of marine algae in neurodegenerative diseases. Int. J. Mol. Sci..

[20] Thomas N.V., Ghafour D.D., Diyya A.S.M., Ismail R.R., Jalal L.K. (2021). Antibacterial effects of the organic crude extracts of freshwater algae of sulaymaniyah, kurdistan region, iraq. J. Med. Plants Res..

[21] Frassini R., Steffens D., Moura S., Aguzzoli C., Martins A.P., Colepicolo P., Fujii M.T., Yokoya N.S., De Pereira C.M.P., Phillipus A.C. (2022). Desmarestia anceps montagne (phaeophyceae) against colorectal cancer cells: Cytotoxic activity and proapoptotic effects. Adv. Biol. Chem..

[22] Carpena M., García-Pérez P., García-Oliveira P., Chamorro F., Otero P., Lourenço-Lopes C., Cao H., Simal-Gandara J., Prieto M. (2023). Biological properties and potential of compounds extracted from red seaweeds. Phytochem. Rev..

[23] Pradhan B., Ki J.-S. (2023). Biological activity of algal derived carrageenan: A comprehensive review in light of human health and disease. Int. J. Biol. Macromol..

[24] Gordalina M., Pinheiro H.M., Mateus M., da Fonseca M.M.R., Cesário M.T. (2021). Macroalgae as protein sources—A review on protein bioactivity, extraction, purification and characterization. Appl. Sci..

[25] Cunha S.A., Pintado M.E. (2022). Bioactive peptides derived from marine sources: Biological and functional properties. Trends Food Sci. Technol..

[26] Ismail M.M., Alotaibi B.S., El-Sheekh M.M. (2020). Therapeutic uses of red macroalgae. Molecules.

[27] Hentati F., Tounsi L., Djomdi D., Pierre G., Delattre C., Ursu A.V., Fendri I., Abdelkafi S., Michaud P. (2020). Bioactive polysaccharides from seaweeds. Molecules.

[28] Zhang L.-X., Cai C.-E., Guo T.-T., Gu J.-W., Xu H.-L., Zhou Y., Wang Y., Liu C.-C., He P.-M. (2011). Anti-cancer effects of polysaccharide and phycocyanin from porphyra yezoensis. J. Mar. Sci. Technol..

[29] Anand J., Sathuvan M., Babu G.V., Sakthivel M., Palani P., Nagaraj S. (2018). Bioactive potential and composition analysis of sulfated polysaccharide from acanthophora spicifera (vahl) borgeson. Int. J. Biol. Macromol..

[30] Shao P., Chen X., Sun P. (2013). In vitro antioxidant and antitumor activities of different sulfated polysaccharides isolated from three algae. Int. J. Biol. Macromol..

[31] Bhuyan P.P., Nayak R., Patra S., Abdulabbas H.S., Jena M., Pradhan B. (2023). Seaweed-derived sulfated polysaccharides; the new age chemopreventives: A comprehensive review. Cancers (Basel).

[32] Rifi M., Radwan Z., AlMonla R., Fajloun Z., Sabatier J.M., Kouzayha A., El-Sabban M., Mawlawi H., Dassouki Z. (2022). The lebanese red algae jania rubens: Promising biomolecules against colon cancer cells. Molecules.

[33] Torres P.B., Nagai A., Jara C.E.P., Santos J.P., Chow F., Santos D.Y.A.C.d. (2021). Determination of sulfate in algal polysaccharide samples: A step-by-step protocol using microplate reader. Ocean Coast. Res..

[34] Ji C.F., Ji Y.B., Meng D.Y. (2013). Sulfated modification and anti-tumor activity of laminarin. Exp. Ther. Med..

[35] Mouradi A., Chikhaoui-Khay M., Akki S.A., Akallal R., Hrrimle I., Givernaud T. (2006). Analyse structurale des fractions polysaccharidiques extraites de laparoi cellulaire d’hypnea musciformis (rhodophyceae, gigartinales). Afr. Sci. Rev. Int. Sci. Technol..

[36] Amimi A., Mouradi A., Givernaud T., Chiadmi N., Lahaye M. (2001). Structural analysis of *gigartina pistillata carrageenans* (*gigartinaceae*, *rhodophyta*). Carbohydr. Res..

[37] Rochas C., Lahaye M., Yaphe W. (1986). Sulfate content of carrageenan and agar determined by infrared spectroscopy. Bot. Mar..

[38] Martínez-Ruiz M., Martínez-González C.A., Kim D.-H., Santiesteban-Romero B., Reyes-Pardo H., Villaseñor-Zepeda K.R., Meléndez-Sánchez E.R., Ramírez-Gamboa D., Díaz-Zamorano A.L., Sosa-Hernández J.E. (2022). Microalgae bioactive compounds to topical applications products—A review. Molecules.

[39] Kumari A., Garima, Bharadvaja N. (2023). A comprehensive review on algal nutraceuticals as prospective therapeutic agent for different diseases. 3 Biotech..

[40] Lajili S., Ammar H.H., Mzoughi Z., Amor H.B.H., Muller C.D., Majdoub H., Bouraoui A. (2019). Characterization of sulfated polysaccharide from laurencia obtusa and its apoptotic, gastroprotective and antioxidant activities. Int. J. Biol. Macromol..

[41] Matloub A.A., Aglan H.A., El Souda S.S.M., Aboutabl M.E., Maghraby A.S., Ahmed H.H. (2016). Influence of bioactive sulfated polysaccharide-protein complexes on hepatocarcinogenesis, angiogenesis and immunomodulatory activities. Asian Pac. J. Trop. Med..

[42] Zhao D., Xu J., Xu X. (2018). Bioactivity of fucoidan extracted from laminaria japonica using a novel procedure with high yield. Food Chem..

[43] Jose G.M., Kurup G.M. (2017). Sulfated polysaccharides from padina tetrastromatica arrest cell cycle, prevent metastasis and downregulate angiogenic mediators in hela cells. Bioact. Carbohydr. Diet. Fibre.

[44] Ghannam A., Murad H., Jazzara M., Odeh A., Allaf A.W. (2018). Isolation, structural characterization, and antiproliferative activity of phycocolloids from the red seaweed laurencia papillosa on mcf-7 human breast cancer cells. Int. J. Biol. Macromol..

[45] Gheda S., El-Sheekh M., Abou-Zeid A. (2018). In vitro anticancer activity of polysaccharide extracted from red alga jania rubens against breast and colon cancer cell lines. Asian Pac. J. Trop. Med..

[46] Morais A.M., Alves A., Kumla D., Morais R.M. (2021). Pharmaceutical and biomedical potential of sulphated polysaccharides from algae. Polysaccharides of Microbial Origin: Biomedical Applications.

[47] Kanaan H., Belous O., Chokr A. (2015). Diversity investigation of the seaweeds growing on the lebanese coast. J. Mar. Sci. Res. Dev..

[48] Sunarwidhi Prasedya E., Miyake M., Kobayashi D., Hazama A. (2016). Carrageenan delays cell cycle progression in human cancer cells in vitro demonstrated by fucci imaging. BMC Complement. Altern. Med..

[49] Ling N. (2012). Growth inhibition and cell cycle arrest of kappa-selenocarrageenan and paclitaxel on hepg2 cells. Adv. Mater. Res..

[50] Raman M., Doble M. (2015). Κ-carrageenan from marine red algae, kappaphycus alvarezii–a functional food to prevent colon carcinogenesis. J. Funct. Foods.

[51] Liu Z., Gao T., Yang Y., Meng F., Zhan F., Jiang Q., Sun X. (2019). Anti-cancer activity of porphyran and carrageenan from red seaweeds. Molecules.

[52] Wali A.F., Majid S., Rasool S., Shehada S.B., Abdulkareem S.K., Firdous A., Beigh S., Shakeel S., Mushtaq S., Akbar I. (2019). Natural products against cancer: Review on phytochemicals from marine sources in preventing cancer. Saudi Pharm. J..

[53] Alves C., Silva J., Pinteus S., Gaspar H., Alpoim M.C., Botana L.M., Pedrosa R. (2018). From marine origin to therapeutics: The antitumor potential of marine algae-derived compounds. Front. Pharmacol..

